# Modelled drift patterns of fish larvae link coastal morphology to seabird colony distribution

**DOI:** 10.1038/ncomms11599

**Published:** 2016-05-13

**Authors:** Hanno Sandvik, Robert T. Barrett, Kjell Einar Erikstad, Mari S. Myksvoll, Frode Vikebø, Nigel G. Yoccoz, Tycho Anker-Nilssen, Svein-Håkon Lorentsen, Tone K. Reiertsen, Jofrid Skarðhamar, Mette Skern-Mauritzen, Geir Helge Systad

**Affiliations:** 1 Centre for Biodiversity Dynamics, Department of Biology, Norwegian University of Science and Technology, 7491 Trondheim, Norway; 2 Department of Natural Sciences, Tromsø University Museum, PO Box 6050 Langnes, 9037 Tromsø, Norway; 3 Norwegian Institute for Nature Research, FRAM—High North Research Centre for Climate and the Environment, 9296 Tromsø, Norway; 4 Institute of Marine Research and Hjort Centre for Marine Ecosystem Dynamics, PO Box 1870 Nordnes, 5817 Bergen, Norway; 5 Department of Arctic and Marine Biology, University of Tromsø, PO Box 6050 Langnes, 9037 Tromsø, Norway; 6 Norwegian Institute for Nature Research, PO Box 5685 Sluppen, 7485 Trondheim, Norway

## Abstract

Colonial breeding is an evolutionary puzzle, as the benefits of breeding in high densities are still not fully explained. Although the dynamics of existing colonies are increasingly understood, few studies have addressed the initial formation of colonies, and empirical tests are rare. Using a high-resolution larval drift model, we here document that the distribution of seabird colonies along the Norwegian coast can be explained by variations in the availability and predictability of fish larvae. The modelled variability in concentration of fish larvae is, in turn, predicted by the topography of the continental shelf and coastline. The advection of fish larvae along the coast translates small-scale topographic characteristics into a macroecological pattern, *viz.* the spatial distribution of top-predator breeding sites. Our findings provide empirical corroboration of the hypothesis that seabird colonies are founded in locations that minimize travel distances between breeding and foraging locations, thereby enabling optimal foraging by central-place foragers.

## Abstract (web summary)

Seabirds breed in high density colonies, but the factors determining colony position aren't clear. Here, Sandvik *et al*. show that small-scale coastal topography is related to likely variation in fish larval abundance, which predicts the distribution of seabird colonies along the Norwegian coast.

## Graphical abstract

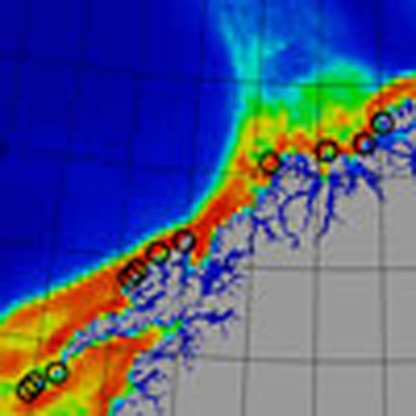

Advantages and disadvantages of colonial breeding are associated with social interactions, predator avoidance and food acquisition (exchange of information), for which selection pressures vary across species^1^,^2^,^3^. Whereas 13% of all birds are colonial breeders, ∼98% of the some 330 seabird species breed in colonies comprised of often very dense aggregations of breeding territories^4^,^5^. Possible explanations include the minimization of travel distances between the nest and foraging locations (geometrical model^1^,^6^), enhanced food finding efficiency through information transfer (information centre hypothesis^7^), limited nest-site availability^1^, predator avoidance^8^ and mate-choice mechanisms^2^.

The distance between neighbouring colonies is often negatively related to their size^9^, indicating a regulating role of intraspecific competition and emphasising the importance of food availability^10^,^11^,^12^,^13^. The mechanisms behind dispersal of individuals from and their immigration into already existing colonies are increasingly understood^14^,^15^. However, the question remains what determined the placement of seabird colonies along a coast in the first place. Whereas recent colonization of empty spaces may have been determined by human-induced habitat changes^16^, the initial colonization of an area can be expected to be linked to the predictability of habitat quality^17^. Habitat quality may be rather straightforward to quantify in terrestrial ecosystems^18^,^19^,^20^, but this is not as easily accomplished for species feeding on oceanic prey^21^,^22^. Of the main factors contributing to nesting habitat suitability, features such as substrate and the absence of terrestrial predators are predictably determined by a site's physical properties, whereas food availability in the local marine environment is much less predictable in time and space. The formation and maintenance of large seabird colonies in relation to semi-stable physical oceanic phenomena, such as upwellings, fronts, gyres and polynyas, nonetheless indicate that there may, under some conditions, be a certain degree of predictability of food supply within the foraging range of seabirds^1^,^23^,^24^. Until now, however, no studies have documented more than theoretical associations between food availability and the distribution of seabird colonies, and little data exist to evaluate the predictions empirically^21^,^22^,^25^,^26^.

We carried out a quantitative test of the association between modelled food availability/predictability and colony locations, using empirical data from the coast of Northern Norway (66–71°N). More than 90% of the two million pairs of Norwegian cliff-nesting seabirds (mainly Atlantic puffin *Fratercula arctica*, black-legged kittiwake *Rissa tridactyla* and common guillemot *Uria aalge*) occur north of 66°N (Fig. 1) (ref. 27). This distribution has been attributed to favourable physical oceanographic conditions linked to the northward-flowing Norwegian Coastal Current (NCC) and to bathymetry, because the exceptional marine productivity at the continental shelf break is close to the coastline (Fig. 1) (refs 27, 28). Such productivity is essential to sustain the large numbers of breeding seabirds, whose foraging range is constrained by flight costs and the energetic demands of raising offspring^29^.

Along the long stretch of Northern Norway's coast—roughly 1,200 km along its shortest line—there are 20 large seabird colonies (here defined as 10,000 breeding pairs or more). As suitable habitat (high cliffs, turf-covered islands and promontories that are all but inaccessible to terrestrial predators) is available along almost all the coast, and human disturbance is minimal for most of its parts, nest-site availability is not a factor limiting the distribution of seabird colonies in Northern Norway. We hypothesize that large seabird colonies are located in those areas where large numbers of planktonic organisms, for example, small crustaceans (especially *Calanus finmarchicus*), fish and their spawning products (eggs and larvae), occur predictably within the foraging range of breeding birds during their breeding period. Such organisms are important food items for both seabirds and some of their fish prey (for example, capelin *Mallotus villosus*, 0- and I-group herring *Clupea harengus*, sandeels *Ammodytes* spp.), and an independent quantification of their abundance is thus a potential proxy of food availability for seabirds.

Coupled ocean circulation and biophysical models allow the quantification of seasonally predictable biological production^30^,^31^. Here, we use an ocean circulation model hindcast to quantify the drift of particles, such as eggs and larvae, from fish spawning grounds along the coast and their interaction with the physical environment^32^, providing a proxy of the abundance of ichthyoplankton throughout the study area with a spatial resolution of 4 km^2^. The output of this model has been validated against empirical observations in the Barents Sea^32^,^33^, and the proxy has already proven to be useful by giving novel insights into several aspects of seabird ecology, such as the day-to-day and interannual variation in stress hormones^34^, fledging weight^35^ and population dynamics^36^ of common guillemots.

Using this approach, we relate the positions of seabird colonies directly to the modelled small-scale temporal and spatial variation of plankton along a coastline. Our findings provide an empirical corroboration of the hypothesis that the initial formation of colonies minimizes travel distances between breeding and foraging locations in a marine habitat.

## Results

### Physical correlates of high larval density

Despite the fact that the NCC transports spawning products along the entire Norwegian coast into the Barents Sea (Supplementary Fig. 1a), the larval drift models show clearly that these particles are not uniformly distributed along the coast. Instead, their distribution is rather patchy with widely varying concentrations (Fig. 2a). Physical features explain 39% of the variation in particle density (Supplementary Table 1). This is mainly due to a gradient on a large spatial scale, *viz* a decrease in particle concentration towards the east. Since the particle trajectories spread out as they approach the Barents Sea, the maximum is reached at 68° 15′ N, where the currents are funnelled round the Lofoten Islands by the narrowing continental shelf.

The smaller-scale patchiness is caused by the interaction between the NCC and coastal morphology, two aspects of which have been quantified: in areas where the continental shelf is narrow, the NCC is constricted near the coast, so that bathymetry explains 7% of the variation in particle density. A further 10% are explained by the structure of the coastline. This is likely because the NCC interacts with bank structures, islets and promontories along the coast, generating stationary eddies that increase the residence time of passive particles such as crustaceans, fish eggs and larvae.

### Relation of seabird colonies to larval density

The existing seabird colonies are located in or close to areas where particle concentrations are higher than the average for the respective coastal segments (Fig. 2a), with the association between seabird colonies and grid cells with high particle abundance being stronger than expected by chance (*P*=0.014). The association between seabird colonies and grid cells with low interannual variability in prey abundance is even stronger (*P*<0.001; Figs 2b and 3a). This means that seabird colonies along the North Norwegian coast are systematically located to give a much better and more stable, that is, predictable, access to suitable food than randomly chosen locations. The importance of low temporal variability in prey abundance is further highlighted by the fact that the colony locations are strongly associated with grid cells that have high minimum food availability across years (*P*=0.013), but less so with grid cells that have high maximum food availability (*P*=0.059; Supplementary Table 2).

Simulations based on fish species rather than generic particles, that is, simulations that take into account the spawning sites, growth patterns and vertical migration behaviour of particular species, showed divergent results. Herring larvae alone did not yield a significant association with colony sites (*P*=0.36), whereas simulations based on cod (*Gadus morhua*) larvae (*P*=0.003) or combinations of both species did (*P*<0.025; Supplementary Table 2). Cod larvae thus produced a model with a much stronger association between larval abundance and colony placement than the generic particle model. A *post-hoc* analysis of the different spawning grounds of cod revealed that this very significant pattern was due mainly to one spawning ground, the Lofoten area (*P*=0.005; Supplementary Table 3). This indicates that any perturbations to cod spawning in this area may have serious consequences for seabirds breeding in and to the north of Lofoten, an area that holds 19 out of the 21 large seabird colonies in Norway. The poorer performance of the herring simulation is explained by the spawning grounds of herring being further south, resulting in considerably lower concentrations of herring than cod larvae along the northern and eastern parts of the coast.

### Significance of foraging distance

The strength of the association between seabird colonies and grid cells with high particle abundance varies depending on the foraging range of the seabirds (Fig. 3b,c). In the initial model, a foraging radius of 10 km is assumed. However, significant associations are obtained for foraging radii of up to 25 km, and associations are strongest for radii between 6 and 8 km (*P*<0.01, Fig. 3b,c). This can be taken to indicate that seabird colonies are usually situated within 5–25 km of productive food patches.

Although recent tracking studies have revealed that several seabirds are capable of foraging at much larger distances from their breeding colony (>400 km)^37^, average trip length under normal conditions is much shorter (<50 km)^38^,^39^. Long trips are costly and are most likely forced upon birds only under unusual food conditions^37^. Our results indicate that seabirds are rather successful in choosing breeding sites that minimize travel distances between food patches and the nest.

### Robustness of results

The association between seabird colonies and particle abundance, which is estimated using randomization tests, holds for a broad range of model parameterizations and is further corroborated by *post-hoc* tests (Fig. 3 and Supplementary Tables 2 and 3), indicating that it is very robust to changes in the background assumptions that had to be made. By modifying some of the initial assumptions, model fit could be substantially improved (all details in Supplementary Table 2), for example, by considering conditions in July alone rather than the average of the breeding season (*P*=0.002), by using a wider definition of suitable coastline (*P*<0.001 for the widest definition) or by using smaller or larger size thresholds for the inclusion of colonies than 10,000 breeding pairs (*P*=0.006 for colonies with at least 5,000 breeding pairs). On the other hand, results were rather insensitive to transformation of larval abundance data (Supplementary Table 2), to the quantile used to represent larval counts (Fig. 3b) or to the weighting function of different foraging distances within the maximum foraging radius (Fig. 3c).

The *post-hoc* tests showed that the association was not merely due to one influential colony location; and that data for a single year of an even more highly resolved larval model (800 m) are compatible with the 4 km model (Supplementary Table 3). When decreasing the grid resolution of the simulations, the association becomes gradually weaker as sample size decreases and the grid cell size exceeds foraging radius; however, even for a fourfold decrease in resolution, the association remains significant for cod (*P*=0.025) and marginally so for the generic particles (*P*=0.051). When considering probabilities for single years, the association between particles and seabird colonies is significant at the 5% level in 23 out of 30 years in the period from 1982 to 2011, the non-significant years do not cluster, and there is no temporal trend in the probabilities (Fig. 3d).

## Discussion

During their breeding season, seabirds are central-place foragers depending on spatially patchy and temporally variable food. According to the geometrical model and optimal foraging theory, such conditions favour the evolution of colonial breeding^6^. Our findings accord with the hypothesis that seabird colonies are established in locations that minimize distances between breeding and foraging locations: colonies are considerably closer to the nearest high-density prey patch than suggested by the potential flight range of seabirds and than expected by chance. Although documented for colonial breeders in terrestrial ecosystems^18^,^19^,^20^, this has been difficult to show in marine ecosystems, presumably because the small-scale distribution of prey items is harder to quantify (but see refs 21, 22).

This analysis has been made possible by a coupled ocean circulation and biophysical model of ichthyoplankton abundance. At the same time, the use of a modelling approach introduces some additional uncertainty, because the empirical validation^32^ is necessarily restricted to a fraction of the area and time period covered by the models. It is also obvious that the findings are open to alternative interpretations, and that the model does not identify all factors relevant for the establishment and maintenance of seabird colonies. There is, for instance, a suspicious absence of seabird colonies in two regions where Fig. 2a suggests high levels of larvae (*viz*, 67.7°–68.7°N and 69.3°–70.1°N). However, the interannual variability of larval abundance is somewhat higher in these regions (0.39±0.12 (quartile coefficient of dispersion (QCD)±s.d.; see the Methods: Simulation: iv), *N*=52 grid cells) than in the surrounding colony locations (0.27±0.02, *N*=4; cf. Fig. 2b). Of course, a number of other causes can be involved in the absence of colonies, including habitat quality, predation pressure, human disturbance and stochastic factors.

One major cost of coloniality is increased competition among foragers at food patches^10^,^11^,^12^,^13^. To sustain coloniality, the food patches must thus be sufficiently large that sharing by foragers is not prohibitively costly, yet they must also be sufficiently scarce and ephemeral that information exchange about their location will result in a higher food intake relative to the alternative strategy of breeding alone^24^. In our case, the areas of temporary food retention are continually resupplied with fish larvae during their drift northwards in the NCC, thus reducing prey depletion and enhancing the food source for seabirds. Areas of particle accumulation may also exhibit high primary production caused by turbulence, which might synergistically improve the foraging conditions for seabird prey.

Although the data on larval abundance used here spanned a 30-year period, the spatial larvae aggregation patterns we have identified are almost certainly more permanent in nature, especially to the degree they are linked to coastal topography and bathymetry. According to historical^40^, archaeological^41^,^42^ and sedimentological evidence^43^,^44^,^45^, seabird colonies are known to have been occupied for hundreds, and even thousands, of years. No such datings are available for Norway. However, the long-term importance of the Lofoten area as a cod spawning area is documented by the fact that this area has exported stockfish at least since the Viking age (that is, for 900 years)^46^. It is therefore possible that the distribution of large colonies along the Norwegian coast constitutes a macroecological pattern that has been stable for a long time, potentially since the end of the last glaciation, *c.* 9,000 years ago.

## Methods

### Oceanography of the study system

Variability in oceanographic features of the Northeastern North Atlantic causes pronounced variations in the distribution and abundance of larvae and juvenile fish in the marine pelagic ecosystem^32^. The physical conditions in the model domain are dominated by the influx of relatively warm Atlantic water through the Faeroe–Shetland Channel^47^, continuing as a two-branch flow, with the outer branch known as the Norwegian Atlantic Slope Current (NASC) along the shelf break (Supplementary Fig. 1a)^48^. On the shelf, the less saline NCC, originating from south of Norway but fed by rivers all along the coast, is trapped between the NASC and the coast^49^. Interannual variations in its physical features affect the dispersal of ichthyoplankton^50^. The frontal zone area near the northern Norwegian shelf break separates the northward-flowing NASC and NCC, and is associated with meso-scale meanders, filaments and eddies^51^. Short-term variability of the flow is forced by variable winds and tide, whereas long-term features are controlled by topography such as banks and troughs.

### Larval drift model

By using a daily hydrodynamic model archive with 4 km × 4 km horizontal resolution (vertical resolution into 32 sigma-levels; baroclinic time step 160 s; barotropic time step 5 s), we simulate the horizontal dispersion of passive particles along the Norwegian coast. The ocean circulation model provides a 30-year time series of hydrography from 1982 to 2011 stored on a daily basis^33^. This spatial resolution is sufficient in order to resolve the eddy field adequately^52^. An individual-based larval drift model was used to advect passive particles from potential spawning areas along the entire Norwegian coast. A full description of the features of this model has been provided by Myksvoll *et al*.^35^ The larval drift model, as well as the ocean circulation model underlying it, have been validated empirically using observations from the Norwegian and Barents Sea^32^,^33^.

**Figure 1:**
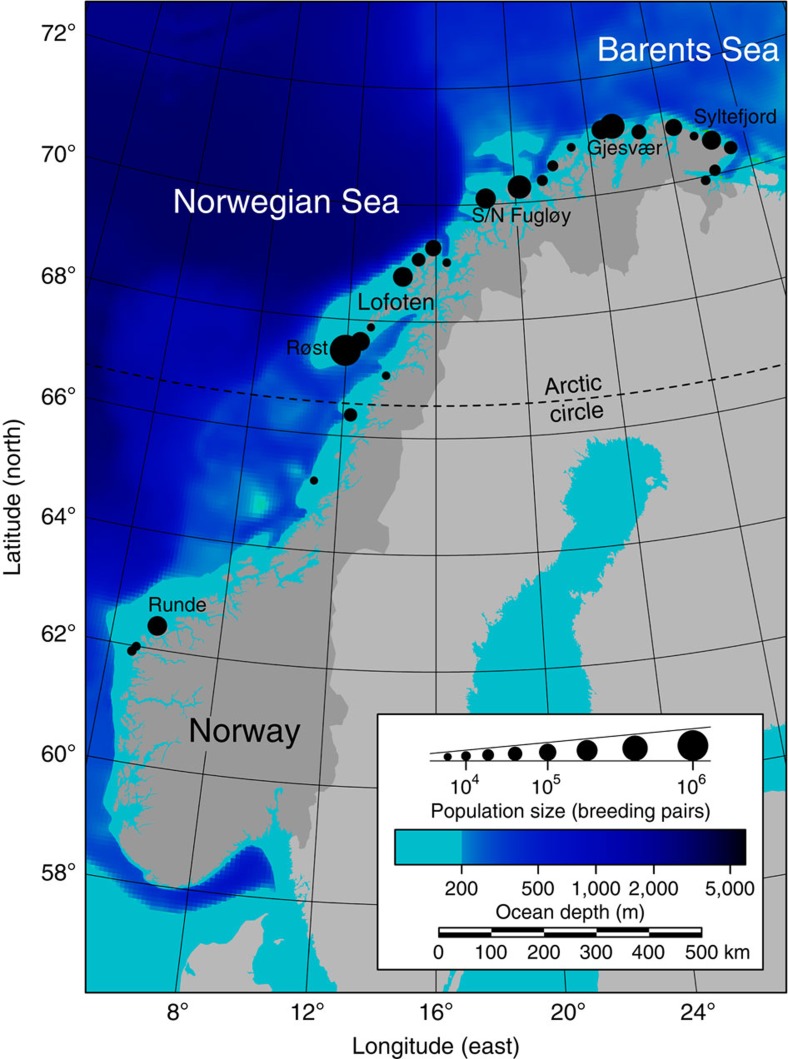
Map of Norway displaying the largest seabird colonies and sea bottom topography. All colonies with more than 5,000 breeding pairs of cliff-nesting species are shown. The entire continental shelf (turquoise) north of 61°N was used as initiation area of the generic larval drift model.

**Figure 2:**
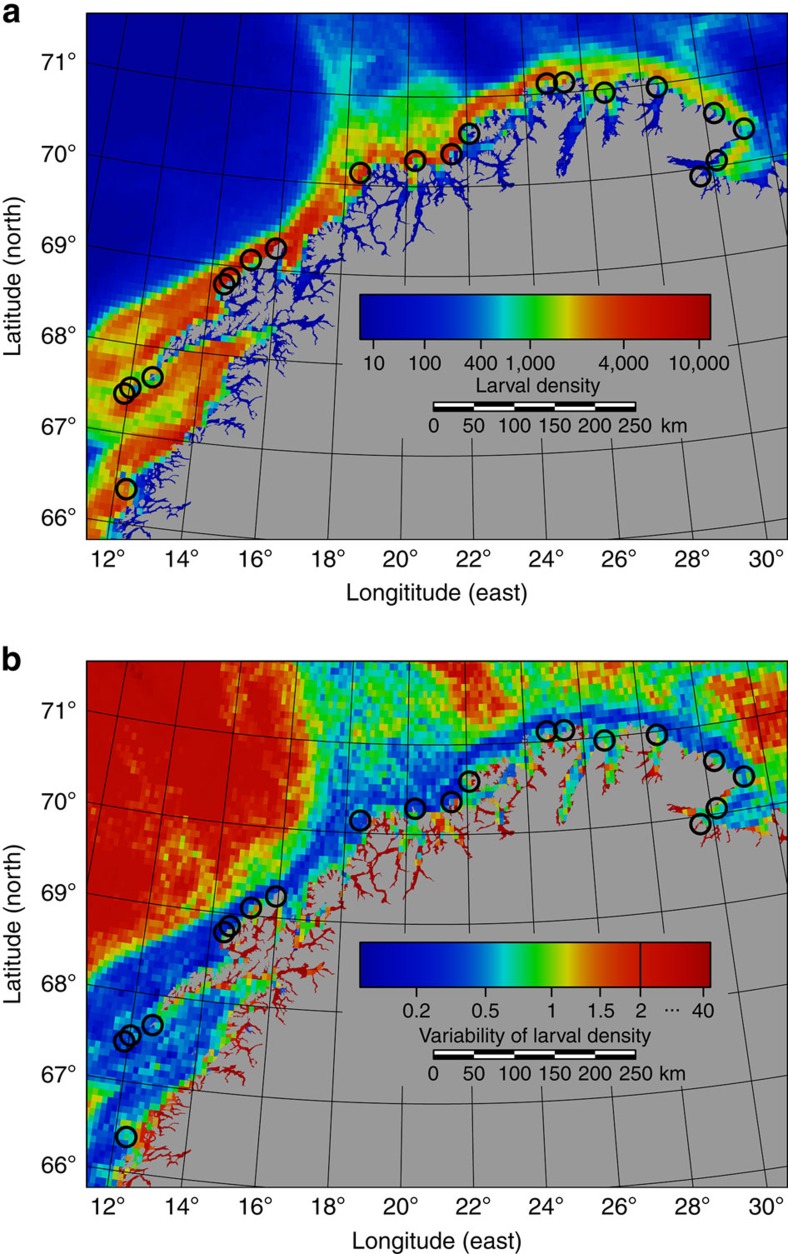
Association between abundance of fish larvae and the location of seabird colonies along the coast of Northern Norway. Circles indicate foraging ranges with radii of 10 km around the 20 largest seabird colonies (at least 10,000 breeding pairs). (**a**) Map of seabird colonies and sites of high modelled larval abundance. There are more high-abundance grid cells within the circles than expected by chance (*P*=0.014). (**b**) Map of seabird colonies and sites of high predictability (low variability) of modelled larval density. Variability is measured as quartile coefficients of dispersion. There are more low-variability grid cells within the circles than expected by chance (*P*=0.0003).

In a generic model, the entire continental shelf (that is, all grid points with bottom depth less than 200 m; Fig. 1) from 61°N and northwards were used as initial release locations for passive particles (6,156 grid points in total). One particle was released per grid point per day from 1 March to 30 April, adding up to 369,360 passive particles in total. After release, the particles drifted freely with the ocean currents for 3 weeks, representing the fish egg stage, followed by a phase with vertical migration depending on swimming capability and light availability, representing the fish larval stage^53^. Although designed to represent the physiology and behaviour of fish larvae, the model also captures the drift of other, more or less passively drifting organisms, such as crustaceans or fish at the small fry stage.

Particle abundance was interpolated to a gridded data set with a latitudinal resolution of 0.075° (*c.* 8.3 km) and a longitudinal resolution of 0.150° (*c.* 5.4 to 6.8 km). The same grid was used as the basis for aggregation of seabird numbers and for simulation (*cf.* Supplementary Fig. 1b).

In addition to the generic model, we run two models for specific target species, *viz* Northeast Arctic cod and Norwegian spring-spawning herring. The cod and herring spawning areas overlap with the original release areas in the generic model, but they consist of several separated areas of limited geographical extent (Supplementary Fig. 1c)^54^,^55^. The cod larvae model includes a three weeks long free drifting stage before the eggs hatch into larvae with vertical migration^53^. The herring larvae model only includes the larval stage, as herring have adhesive benthic eggs. The models released a total of 94,500 cod eggs and 230,400 herring eggs from 1 March to 30 April.

### Coastal morphology

The near-shore accumulation of particles is partly governed by physical properties of the coast. We quantified two aspects of coastal morphology for each coastal grid cell, *viz* the width of the continental shelf (bathymetry) and the degree of protrusion of the coastline (topography). Width of the continental shelf was defined as the shortest distance between the points of the coastline within the grid cell and the 200-m isobath (depth-contour), rounded to the nearest kilometre (*cf.* Fig. 1).

Protrusion was quantified by fitting a straight line through portions of coastline as follows: separately for each grid cell, other grid cells within a 40-km radius were considered, sampling from each grid cell the point protruding most towards the ocean (Supplementary Fig. 1d). The straight line was fitted to minimize squared distances for this sample of points (major axis regression), excluding the focus cell. The shortest distance (km) of the most protruding point in the focus cell to this line is referred to as the degree of protrusion of this grid cell. Headlands and outlying islands are characterized by positive protrusions, bays and fjords by negative protrusions. This algorithm could not be unambiguously applied to the southernmost grid cell of the Lofoten archipelago and the islands southwest of Lofoten (Røst, Værøy and others; in total, 9 grid cells, hereafter referred to as ‘ambiguous'; *cf.* Supplementary Fig. 1d). In these cases, protrusion was defined as the distance from the nearest coastal point.

The effect of coastal morphology on particle density was investigated using linear models. Model selection was based on Akaike's Information Criterion. To account for ambiguities regarding protrusion, models were rerun excluding ambiguous grid cells. To correct for spatial autocorrelation, *P*-values were obtained using randomization tests (see below, ‘Spatial autocorrelation'). The proportion of randomized linear models obtaining an equal or larger *R*^2^ than the original linear model was used as a corrected *P*-value.

### Seabird colony data

The position and size of all seabird colonies north of 66°N that at any one time comprised 1,000 or more pairs were extracted from the Norwegian seabird database (http://www.seapop.no; *cf.* Supplementary Table 4). Entries in this database date back to the mid-twentieth century. Species included in the counts were cliff-nesting seabirds known to feed at least partly on fish larvae or the forage fish predators of larvae, that is, razorbill (*Alca torda*), Atlantic puffin (*Fratercula arctica*), northern fulmar (*Fulmarus glacialis*), black-legged kittiwake (*Rissa tridactyla*), common guillemot (*Uria aalge*) and Brünnich's guillemot (*Uria lomvia*). Apart from the fulmar that may forage far out at sea^56^ (but whose breeding numbers are small in Norway), these are species with, under normal conditions, foraging ranges of *c.* 20–50 km from the nest^38^,^39^, that is, considerably larger than the dimension of grid cells.

A grid cell of the larval drift model was considered to contain a seabird colony if the maximum sum of breeding pairs of all species within the grid cell at any point in time was at least 10,000 (but other population thresholds were also considered; see ‘Simulation'). For each grid cell, we considered the maximum overall population count available across years, rather than the count from a specific year, because we were interested in the position of colonies in a long-term perspective, independent of short-term variation in the size of breeding populations. This procedure yielded a sample of 20 seabird colonies of at least 10,000 breeding pairs.

### Spatial autocorrelation

The spatial autocorrelation of observations introduces a statistical dependence that precludes the use of standard tests. Probabilities were therefore estimated using randomization tests, that is, a Monte Carlo method involving the repeated random reordering of the data. Based on at least 100,000 replications, probabilities were defined as the proportion of reordered data sets that obtained a test statistic as extreme as, or more extreme than, the one observed.

As the former method may not remove all covariation between variables, it was supplemented by the toroidal-shift method as a *post-hoc* test. This test entails shifting or rotating one point pattern against the other, while leaving both point patterns in their original order^57^,^58^. This was accomplished by shifting grid cells along the coastline, treating the latter as a closed ring (torus), that is, by connecting the two ends of the coastline. To avoid edge effects in models involving colony locations, both ends of the coastline were defined as the grid cell placed midway between the ultimate and penultimate colonies, reducing the number of colonies included by 2. The sample size of this test equals the number of coastal grid cells.

### Simulation

Simulations were used in order to estimate the probability that randomly distributed seabird colonies would have equally good or better access to food particles than actual seabird colonies. Each simulation consisted of a randomization test that randomly defined *n* different coastal grid cells as seabird colonies (where *n* is the number of actual colonies of a certain size in Northern Norway), aggregated larval abundance within a certain feeding radius around each colony, summed the *n* values to obtain the total aggregation, and compared this value to the total aggregation around the actual colonies. This procedure was repeated 1,000,000 times for the initial model.

**Figure 3:**
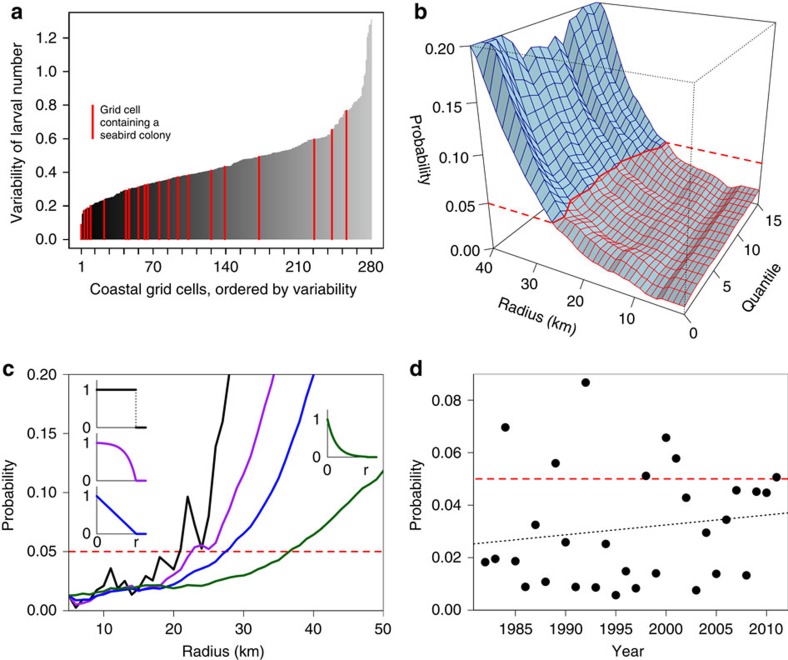
Results of simulations and relaxation of the assumptions on which the initial model is based. (**a**) Histogram of the 280 grid cells along the coast, sorted by increasing variability of larval abundance (as measured by the quartile coefficient of dispersion). Grid cells containing the 20 largest seabird colonies, highlighted as red bars, are concentrated in the left part of the histogram, that is, they have significantly lower variability in larval abundance than randomly chosen grid cells. (If uniformly distributed, the 20 red bars would lie exactly between the 21 ticks of the *x*-axis.) (**b**–**d**) The panels report the probability that a sample of 20 randomized colony positions obtains a better availability of fish larvae than the actual positions of the 20 largest seabird colonies in Northern Norway. (Each point in the parameter space is based upon simulations with 100,000 replicates. The portion of the parameter space below the red line is statistically significant at the 5% level). (**b**) Probabilities given different radii of feeding ranges around seabird colonies (relaxation of assumption (vii)) and given different 30-quantiles of particle abundance for each grid cell (relaxation of assumption (iv)). The 0th 30-quantile corresponds to the minimum particle density within the 30-year period, the 15th 30-quantile to the median. The initial model uses a radius of 10 km and the 3rd 30-quantile. (**c**) Probabilities given different feeding radii (*r*) and different weighting schemes of particles within the feeding radius specified (relaxation of assumption (viii)): none-or-all threshold (black), convex weighting function (purple), linear weighting function (blue), concave weighting function (green). Small panels visualize the weighting functions. The initial model uses a linear weighting scheme. (**d**) Probabilities for single years. There is no significant trend in the probabilities (dotted line; slope 0.00038±0.00047, *R*^2^=0.02, *P*=0.42).

Before simulation, certain assumptions had to be made, involving (i) the prey species modelled; (ii) the months considered; (iii) how to quantify larval abundance; (iv) how to take the inter-annual variability of larval abundance into account; (v) the selection of grid cells deemed suitable for seabird colonies; (vi) the size threshold for actual colonies included; (vii) the radius of the foraging range and (viii) the weighting of larval numbers within the feeding range. The optimal parameterization and its importance for the results were unknown in advance. Therefore, the initial model was built upon a reasonable set of *a priori* assumptions, all of which were relaxed in separate analyses (*cf.* Supplementary Table 2), each based on simulations with 100,000 replicates:

- The initial model was based on generic particles rather than specific fish species. This means that the model captures features common to all planktonic prey items. It was contrasted with separate simulations based on cod larvae, herring larvae and combinations of these two. In one combined model, fish larvae abundance in each grid cell was defined as the sum of cod and herring larvae in each grid cell, scaled by the overall geometric mean for each species. In the other combinations, herring was considered up to a latitude of 68° 30′ N or 69° 30′ N, respectively, and cod north of these thresholds.
- Output from larval drift models was extracted for May, June and July of each year, which constitute the main breeding season of the seabirds considered. The initial model is based on the mean larval abundance for this 3-month period for each year. Single months were also tested.
- The larval abundances across grid cells were heavily skewed, with many grid cells having low abundances, and a few cells with very high abundances. A selection of grid cells containing one of the latter cells could theoretically bias the simulations, if the larval abundance in this cell overshadows the abundances of the remaining grid cells. To avoid this potential bias, the initial model was based on log-transformed larval abundances. This model was contrasted with (a) simulations using untransformed larval abundances and (b) simulations that merely considered the ordering of grid cells (that is, grid cells were ranked in the order of their larval abundances, and ranks, rather than larval abundances, were compared).
- We hypothesize that optimal colony sites are those with high mean abundance and low inter-annual variability in abundance of larvae. As a measure that captures aspects of both central tendency and variability, we used the 1st decile (3rd 30-quantile) of larval abundances for each grid cell. This means that, for each grid cell and in 9 out of 10 years, larval abundances were equal to or higher than the specific value identified. This simulation was contrasted with others in which larval abundance was represented by 16 quantiles from the minimum (0th 30-quantile) to the median (15th 30-quantile) of the 30 annual estimates for each of the grid cells (*cf.* Fig. 3b). In addition, the arithmetic and geometric mean, the maximum value and three measures of variability were tested explicitly, *viz*, the variance, the coefficient of variation (CV=standard deviation/mean) and the QCD (QCD=interquartile range/median). The latter is more robust than the CV when distributions are skewed, and results reported for variability are therefore based on the QCD.
- The result of simulation depends on the selection of coast cells deemed suitable. We therefore produced four sets of grid cells, progressively omitting cells that did not have direct contact with oceanic cells or were placed inside fjords or sounds (Supplementary Fig. 1b). Our main results are reported for the most conservative selection of grid cells (that is, the smallest set). Grid cells outside the outermost colonies (that is, grid cells south/west of the southernmost actual colony on the Norwegian Sea coast and south/east of the southeasternmost actual colony on the Barents Sea coast) were disregarded.
- In the initial model, the threshold for actual colonies included was arbitrarily set at ≥10,000 breeding pairs (20 colonies). In addition, we used other thresholds for the inclusion of actual seabird colonies, *viz*, ≥5,000 pairs, ≥20,000 pairs, ≥50,000 pairs and ≥100,000 pairs, resulting in samples of 9 to 27 colonies (Fig. 1 and Supplementary Fig. 1b).
- Different seabird species have different foraging radii, and foraging ranges reported in the literature are likely to be greater than the distances preferred by the birds (assuming that seabirds are selected to minimize the energetic costs of flight). We therefore had no *a priori* estimate of what the relevant radius might be, and assumed 10 km as a reasonable first guess. In addition, radii of foraging range were varied by 1 km between 5 and 50 km (*cf.* Fig. 3b).
- Different assumptions can be made about how seabirds choose between prey patches in the vicinity of their breeding colony. It is likely that prey close to the colony is preferred over distant prey, but the shape of this preference function has not been quantified. In the initial model, we assume that the degree of preference decreases linearly from 100% at a distance of 0 to 1% at the maximum allowed feeding distance, by weighting larval numbers accordingly. Simulations based on this assumption were contrasted with models with alternative weighting schemes, *viz*., a threshold shape (all prey patches within the feeding radius are weighted equally), a concave and a convex shape (both based on exponential functions reaching 1% at the predefined maximum radius; see Fig. 3c for details).

### *Post-hoc* tests

Based on the results obtained for the initial model and the set of models relaxing its assumptions, a number of further tests were devised (*cf.* Supplementary Table 3). Each of these *post-hoc* tests was based on simulations with 100,000 replicates.

Exclusion of the most influential colony. To ensure that the patterns found are not merely driven by one colony with extremely high larval numbers in its vicinity, the most influential colony was excluded. This was done for the initial model as well as the model of particle variability (QCD) and the model for cod larvae. In each case, the colony that obtained the highest (in case of variability, lowest) number of particles was identified, and the respective grid cell removed from the simulation.

Higher-resolution larval drift model. A sensitivity test was performed using an ocean circulation model with higher resolution (horizontal resolution 800 m × 800 m; vertical resolution into 35 sigma-levels; baroclinic time step 60 s; barotropic time step 1 s; output fields of model variables stored on hourly basis)^59^,^60^. As it was not feasible to process data for 30 seasons at this resolution, the model was run for cod in a single year (2010), the results were interpolated to the same grid as the original data set, and contrasted with the results of the 4-km model for the same fish species and year.

Lower-resolution coastal grid. To ensure that the associations found do not critically depend on the grid chosen, we varied the grid scale by collapsing neighbouring grid cells into larger cells and base the simulations on the new grid obtained. This was done for the generic model and the cod model. We collapsed pairs of grid cells (thus obtaining a latitudinal resolution of 0.075° and a longitudinal resolution of 0.300°) and quadruples of grid cells (thus obtaining a latitudinal resolution of 0.15° and a longitudinal resolution of 0.30°). As there are two different ways to collapse pairs and four different ways to collapse quadruples, all versions were simulated (each 100,000 times), and the average probability is reported. Averaging probabilities is adequate in this case, because each probability expresses a proportion (*viz*, the fraction of simulations that obtained equal or higher larval counts around randomized colonies than around actual colonies).

Toroidal-shift method. The toroidal-shift method (see above, ‘Spatial autocorrelation') was used as an alternative test that takes the spatial autocorrelation pattern of the variables into account. It was applied to the initial model, the model of particle variability (QCD) and the model for cod larvae. As the sample size of this test is the number of grid cells rotated (*N*=260), the *P*-value cannot be lower than 0.004.

Separate analyses of cod spawning grounds. As the distribution of cod larvae gave an even better fit to seabird colonies than the distribution of generic particles, and because the spawning grounds of cod are well known, we quantified the relative importance of the latter. Spawning grounds were aggregated in seven areas, and tested separately.

Coastal characteristics. As both fine-scale measures of coastal topography (protrusion, breadth of continental shelf; *cf.* ‘Coastal topography' above) turned out to be important for the distribution of particles along the coast, we quantified their direct importance for the location of seabird colonies. Simulations were carried out excluding the grid cells with ambiguous definitions of protrusion (see the section ‘Coastal morphology').

Single years. Finally, each year of the 30-year-period was tested separately. This was done in order to ensure that the results were not driven by a few anomalous years (*cf.* Fig. 3d).

### Data availability:

Data used in analyses are archived at the Dryad Digital Repository (doi:10.5061/dryad.3jr62).

## Additional Information

**How to cite this article:** Sandvik, H. *et al*. Modelled drift patterns of fish larvae link coastal morphology to seabird colony distribution. *Nat. Commun.* 7:11599 doi: 10.1038/ncomms11599 (2016).

## Supplementary Material

Supplementary Information Supplementary Figure 1 and Supplementary Tables 1-4
