# Supplementary material for: Modelled drift patterns of fish larvae link coastal morphology to seabird colony distribution
Source: Nat Commun. 2016 May 13;7:11599. doi: 10.1038/ncomms11599 (PMC4869253; doi:10.1038/ncomms11599)
Supplement: Supplementary Information — Supplementary Figure 1 and Supplementary Tables 1-4 [file ncomms11599-s1.pdf]

Supplementary Figure

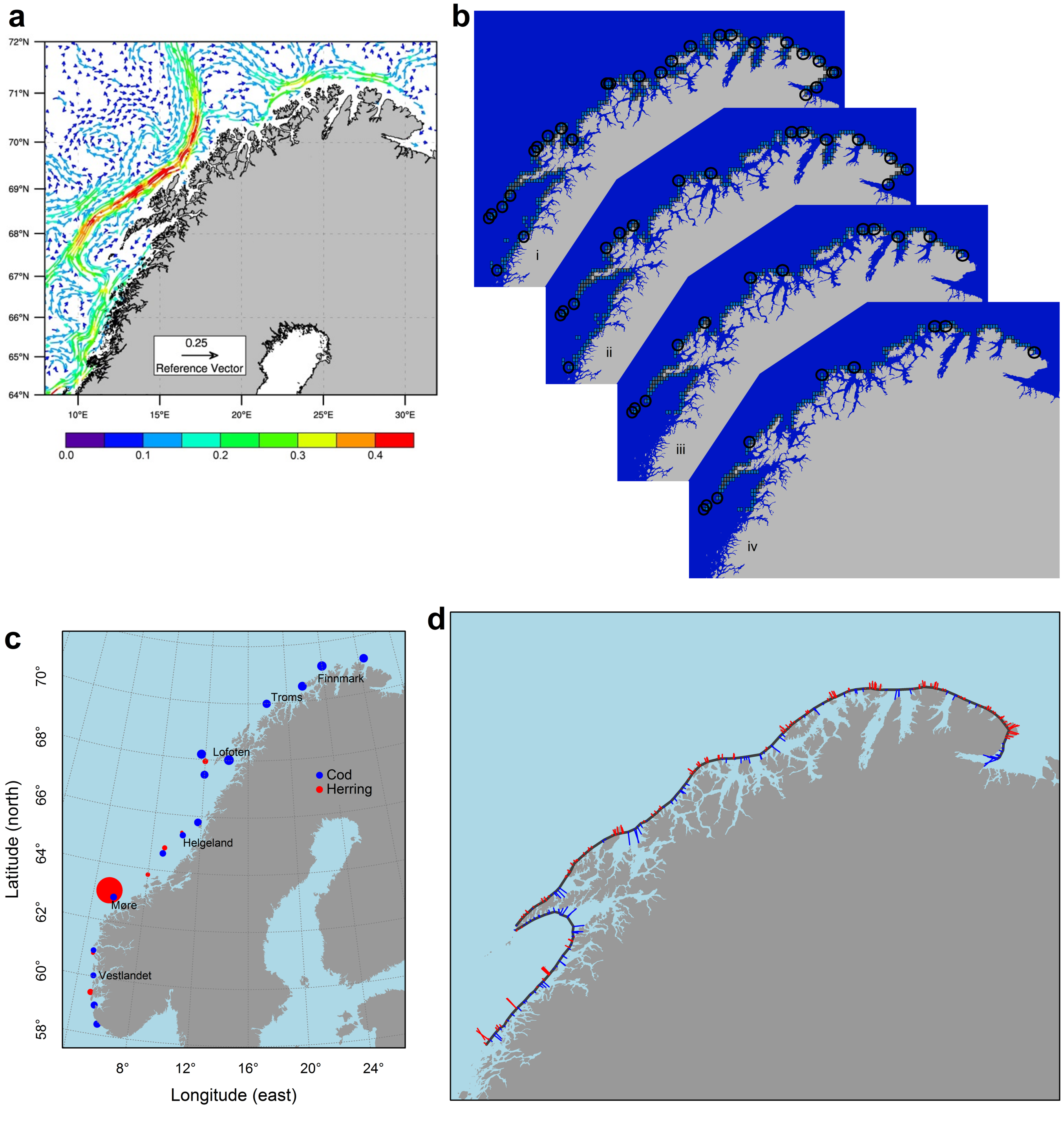

Supplementary Figure 1 | Maps of the Norwegian coast. (**a**) Currents along the coast of Northern Norway. The aggregation of arrows along the shelf break is the Norwegian Atlantic Slope Current (NASC). The Norwegian Coastal Current (NCC) closely follows the coastline. The reference vector and colour codes indicate the velocity of water masses (m s^{-1}). (**b**) Grid cells used in simulations as potential locations for seabird colonies. The panels show the set of actual seabird colonies (circles) when the size threshold is set to (i) 5,000, (ii) 20,000, (iii) 50,000, (iv) 100,000 breeding pairs. Grid cells that are considered as potential positions of seabird colonies are highlighted in different colours and framed in black. The panels illustrate the (i) widest, (ii, iii) intermediate and (iv) narrowest definition of suitable grid cells. The initial model is based on a colony size threshold of 10,000 breeding pairs and the narrowest set of grid cells. (**c**) Spawning areas of cod (blue) and herring (red). These are used as initiation areas for the respective larval drift models. (**d**) The protrusion of each grid cell was estimated as the deviation from the "average coast line" (using major axis regression; bold line). Protrusion is shown as red (positive) or blue lines (negative) for all coastal grid cells.

Supplementary Tables

Supplementary Table 1 | Linear models explaining variation in particle (fish larvae) abundance across grid cells along the North-Norwegian coast

Parameter	Estimate	Standard Error	t [F]	$P_{ord.}$	$P_{rand.}$	$P_{tor.}$	[partial] R^2
**All grid cells (N = 280)**							
Position (SW–NE)	0.098	0.020	5.00	$1 \cdot 10^{-6}$	$2 \cdot 10^{-5}$	0.004	0.056
Position squared	$-4.7 \cdot 10^{-4}$	$6.6 \cdot 10^{-5}$	–7.23	$5 \cdot 10^{-12}$	$1 \cdot 10^{-5}$	0.004	0.117
Protrusion	0.312	0.047	6.71	$1 \cdot 10^{-10}$	$1 \cdot 10^{-5}$	0.004	0.101
Breadth of continental shelf	–0.278	0.051	–5.44	$1 \cdot 10^{-7}$	$1 \cdot 10^{-5}$	0.021	0.066
Model (AIC = 1803.38; ΔAIC = –26.59)			F = 43.31	$< 10^{-15}$	$1 \cdot 10^{-5}$	0.004	0.386
**Excluding ambiguous grid cells (N = 271)**							
Position (SW–NE)	0.092	0.021	4.43	$1 \cdot 10^{-5}$	$3 \cdot 10^{-5}$	0.037	0.045
Position squared	$-4.6 \cdot 10^{-4}$	$6.8 \cdot 10^{-5}$	–6.72	$1 \cdot 10^{-10}$	$1 \cdot 10^{-5}$	0.004	0.103
Protrusion	0.347	0.051	6.80	$9 \cdot 10^{-11}$	$1 \cdot 10^{-5}$	0.004	0.105
Breadth of continental shelf	–0.279	0.052	–5.40	$1 \cdot 10^{-7}$	$1 \cdot 10^{-5}$	0.011	0.066
Model (AIC = 1749.69; ΔAIC = –26.20)			F = 43.18	$< 10^{-15}$	$1 \cdot 10^{-5}$	0.004	0.394

Parameters included are the position of grid cells along the coast (ordered from southwest to northeast; both linear and squared), protrusion from the average coastline (see text for definition) and breadth of continental shelf (distance to the continental slope). Particle abundance is defined as the 1st decile for the period 1982–2011. Based on Box-Cox transformations, particle counts were power-transformed using $\lambda = 0.25$, resulting in residuals that were approximately normally distributed and homoscedastic. Models are presented including and excluding islands to the southwest of the Lofoten archipelago, for which protrusion could not be unambiguously defined. Model selection was based on Akaike's Information Criterion (AIC); the difference to the second best model is indicated in terms of ΔAIC. P-values are provided for the uncorrected tests ($P_{ord.}$), for the randomisation test ($P_{rand.}$, based on 100,000 replicates) and the toroidal-shift method ($P_{tor.}$, based on 280 or 271 position shifts, respectively; for details, see Methods, section "Spatial autocorrelation").

Supplementary Table 2 | Relaxation of assumptions on which the initial model is based

Assumptions	Probability
**(i) Fish species**	
Generic model	*0.01382*
Cod larvae	0.00250
Herring larvae	0.35918
Cod and herring larvae combined (average)	0.02339
Cod and herring larvae combined (latitudinal, divided at 68° 30' N)	0.00980
Cod and herring larvae combined (latitudinal, divided at 69° 30' N)	0.01895
**(ii) Months**	
May, June and July (average)	*0.01382*
May	0.01415
June	0.01105
July	0.00238
**(iii) Data transformation**	
None (linear scale)	0.02817
Log-transformation	*0.01382*
Rank order only	0.01828
**(iv) Measures of central tendency and temporal variability**	
Minimum	0.01284
1st decile	*0.01382*
Median	0.01813
Geometric mean	0.01848
Arithmetic mean	0.02372
Maximum	0.05895
Variance	0.14454
Quartile coefficient of dispersion	0.00026
Coefficient of variation	0.00319
**(v) Definition of suitable coastline**	
466 grid cells	0.00003
350 grid cells	0.00166
303 grid cells	0.01008
280 grid cells	*0.01382*
**(vi) Size threshold for seabird colonies**	
≥ 5,000 breeding pairs (27 colonies, 282 grid cells)	0.00560
≥ *10,000 breeding pairs (20 colonies, 280 grid cells)*	*0.01382*
≥ 20,000 breeding pairs (16 colonies, 274 grid cells)	0.01265
≥ 50,000 breeding pairs (12 colonies, 222 grid cells)	0.00976
≥ 100,000 breeding pairs (9 colonies, 222 grid cells)	0.01238
**(vii) Foraging radius**	
10 km	*0.01382*
20 km	0.02226
30 km	0.06528
40 km	0.19969
50 km	0.39362
**(viii) Weighting of foraging distances**	
None (threshold)	0.02102
Linear decrease with distance	*0.01382*
Concave (exponential decrease with distance)	0.01631
Convex (inversion of the concave weighting function)	0.01444

The Table presents results for different (i) fish species, (ii) months, (iii) data transformations, (iv) data representations, (v) definitions of grid cells suitable for seabird colonies, (vi) size thresholds for seabird colonies, (vii) foraging radii and (viii) weighting schemes. The assumptions underlying the initial model are italicised. The probabilities reported (probability of an equal or better match between larval abundance and randomised colony position than actual colony positions) are derived from simulations with 100,000 replications. See Figure 3a–c for the full relaxation of assumptions (iv), (vii) and (viii).
The Table shows that the relationship found between particle abundance and seabird colonies did not hinge on any of the initial model assumptions that had to be made.

Supplementary Table 3 | *Post-hoc* tests of the initial model

Test performed	Probability
**(a) Exclusion of the most influential colony**	
Generic model (Bleiksøya excluded)	0.03271
Variability model (Frugga excluded)	0.00126
Cod model (Bleiksøya excluded)	0.00650
**(b) Higher-resolution larval drift model**	
High resolution (800 m × 800 m; cod, 2010 only)	0.03173
For comparison: default resolution (4 km × 4 km; cod, 2010 only)	0.02519
**(c) Lower-resolution coastal grid**	
Generic model (0.075° × 0.30°; 20 colonies, 199–202 grid cells)	0.04049
Generic model (0.150° × 0.30°; 19–20 colonies, 145–147 grid cells)	0.05127
Cod model (0.075° × 0.30°; 20 colonies, 199–202 grid cells)	0.01320
Cod model (0.150° × 0.30°; 19–20 colonies, 145–147 grid cells)	0.02537
**(d) Toroidal-shift method (*N* = 260)**	
Generic model	0.004
Variability model	0.004
Cod model	0.004
**(e) Separate analyses of cod spawning grounds**	
Vestlandet / south (58–59° N)	1.00000
Vestlandet / north (60–61° N)	0.91994
Møre (63° N)	0.46276
Helgeland (64–66° N)	0.33696
Lofoten (67–69° N)	0.00459
Troms (70° N)	0.08036
Finnmark (71° N)	0.06511
**(f) Coastal characteristics**	
Protrusion of coast (excluding Røst *etc.*; 17 colonies, 271 grid cells)	0.11551
Breadth of continental shelf	0.08415

The characteristics of the initial model were further explored by (a) excluding the most influential colony; (b) comparing with one year (2010) of data from a cod-larvae drift-model using an even higher resolution; (c) investigating the effect of decreasing grid resolution; (d) using the toroidal-shift method to take spatial autocorrelation into account; (e) comparing the importance of different cod spawning areas; (f) investigating the association between seabird colonies and protrusion of grid cells or breadth of the continental shelf. The probabilities reported (probability of an equal or better match between larval abundance and randomised colony position than actual colony positions) are derived from simulations with 100,000 replications [except (d)]. Results in (a), (b), (c) and (d) produce further corroboration of the initial model; (e) highlights the importance of the Lofoten area as a crucial spawning ground for the remainder of the coast; (f) shows that coastal characteristics are not in isolation sufficient to explain the distribution of seabird colonies.

Supplementary Table 4 | Positions and sizes of cliff-breeding seabird colonies in northern Norway

Seabird colony			Number of breeding pairs in maximum year					
Name	Latitude (N)	Longitude (E)	Year	Razorbill	Puffin	Kittiwake	Guillemot	Total
Lovunden	66° 22’	12° 20’	1979	0	36,400	0	0	36,400
Fugløya	67° 04’	13° 48’	1982	0	5,500	0	0	5,500
Røst (SW)	67° 28’	11° 56’	1979	600	1,045,500	100	1,300	1,047,750*
Vedøy	67° 29’	12° 01’	1990	900	391,500	25,000	3,500	421,050†
Værøy	67° 38’	12° 35’	1974	800	70,000	75,000	1,750	147,550
Moskenes	67° 53’	13° 02’	2006	0	0	5,050	0	5,050
Nykvåg	68° 46’	14° 26’	1990	0	165,000	5,800	0	170,800
Frugga	68° 50’	14° 34’	1990	0	15,000	0	0	15,000
Anda	69° 04’	15° 10’	2009	0	35,900	2,000	0	37,900
Bleiksøya	69° 16’	15° 52’	2009	0	80,000	200	500	80,700
Sundsvollsundet	69° 01’	16° 31’	2005	0	0	6,200	0	6,200
Sør-Fugløy	70° 06’	18° 30’	2009	5,000	186,000	0	100	191,100
Store Mekta	70° 07’	18° 33’	1990	0	7,700	0	0	7,700
Nord-Fugløy	70° 16’	20° 14’	1967	10,000	320,000	0	9,000	339,000
Loppa	70° 21’	21° 25’	1983	2,050	14,500	0	350	16,900
Andotten	70° 35’	22° 01’	1983	0	0	16,600	0	16,600
Lille Kamøy	70° 52’	23° 04’	1984	0	5,000	1,200	500	6,700
Hjelmsøya	71° 06’	24° 44’	1974	7,000	20,000	50,000	70,000	147,850‡
Gjesværstappen	71° 08’	25° 20’	1991	2,500	439,100	1,600	600	443,800
Sværholtklubben	70° 58’	26° 41’	1974	0	0	55,000	0	55,000
Omgangsstauran	70° 56’	28° 31’	1981	100	5,000	90,000	1,000	96,100
Kongsøya	70° 43’	29° 28’	2006	0	0	6,650	0	6,650
Syltefjordstauran	70° 35’	30° 18’	1979	100	0	140,000	9,000	149,100
Reinøya	70° 23’	31° 08’	2009	0	1,900	2,000	1,000	5,000§
Hornøya	70° 23’	31° 10’	1983	200	5,000	21,000	8,750	35,300‖
Store Ekkerøy	70° 04’	30° 08’	1979	0	0	20,000	0	20,000
Ranvika	69° 56’	29° 34’	1989	0	0	10,000	0	10,000

The cross-species maximum count for each colony (including counts from other breeding sites within the same grid cell from the same year) is provided together with the year for this count (source: Norwegian seabird database, http://www.seapop.no). Numbers reported are breeding pairs of razorbills, Atlantic puffins ("puffin"), black-legged kittiwakes ("kittiwake") and common guillemots ("guillemot"). Northern fulmars and Brünnich's guillemots were present in some colonies, too, and are indicated by footnotes:

* The total includes 250 pairs of northern fulmars.
† The total includes 150 pairs of northern fulmars.
‡ The total includes 850 pairs of Brünnich's guillemots.
§ The total includes 100 pairs of Brünnich's guillemots.
‖ The total includes 350 pairs of Brünnich's guillemots.
